# Passive Posterior Tibial Subluxation on Routine Knee MRI as a Secondary Sign of PCL Tear

**DOI:** 10.1155/2014/715439

**Published:** 2014-12-22

**Authors:** Andrew J. Degnan, Catherine Maldjian, Richard J. Adam, Christopher D. Harner

**Affiliations:** ^1^Department of Radiology, University of Pittsburgh Medical Center, 3950 Presby South Tower, 200 Lothrop Street, Pittsburgh, PA 15213, USA; ^2^University of Pittsburgh, Pittsburgh, PA 15260, USA; ^3^Department of Orthopedic Surgery, University of Pittsburgh Medical Center, Pittsburgh, PA 15213, USA

## Abstract

The posterior drawer test is an accurate clinical test to diagnose posterior cruciate ligament (PCL), indicating laxity of the PCL that allows posterior tibial translation. This study aimed to determine whether posterior tibial translation relative to the femur on routine MRI could serve as an additional sign of PCL tear. Routine knee MRI in eleven patients (7 males, 4 females) with arthroscopically confirmed isolated PCL tears were reviewed independently by two musculoskeletal radiologists. Measurements of tibial translation were made in the medial and lateral compartments of patients and controls (10 males, 12 females) without clinical or MRI evidence of ligament injury. Significant medial compartment posterior tibial translation was present in patients with PCL tear compared to controls (+2.93 mm versus +0.03 mm, *P* = 0.002) with excellent interobserver agreement (intraclass correlation coefficient (ICC) = 0.94). No significant difference in lateral compartment tibial translation was observed (+0.17 mm versus −0.57 mm, *P* = 0.366) despite excellent interobserver agreement (ICC = 0.96). Posterior tibial translation in the midmedial compartment may be a secondary sign of isolated PCL tear on routine knee MRI with passive extension without manipulation or weight bearing. Additional work in a larger cohort may better address the accuracy of this finding.

## 1. Introduction

Posterior cruciate ligament (PCL) tears can have deleterious long-term consequences and therefore surgical repair has become a more widely utilized treatment option. In the setting of multiligament injuries, arthroscopy for other injuries may reveal occult PCL tears. In the setting of isolated PCL tear where arthroscopy is not performed, PCL tears that are missed clinically might only be detected on MRI. However, discontinuity of the PCL is not always seen on MRI. Therefore, various indirect signs have been invoked to diagnose PCL tears. Several indirect signs of PCL tears have been suggested, including posterior cruciate ligament thickness, ligamentous laxity, and increased intrasubstance signal [1]. The posterior drawer test is the most accurate clinical test to diagnose PCL tears; however, posterior subluxation often cannot be elicited with this maneuver in the acute setting due to soft tissue swelling and pain [2]. The purpose of this study is to determine if there is significant passive posterior translocation of the tibia relative to the femur in patients with isolated PCL tears on routine MR imaging without weight-bearing or manipulation.

## 2. Materials and Methods

### 2.1. Study Participants

Institutional review board approval was obtained. The institutional database for two orthopedic surgeons was searched retrospectively for PCL tears with arthroscopically intact ACL over a 3-year period. Only patients with preoperative MRI were included in the study. A total of 11 patients fulfilled these criteria. For the control group, 22 knee MRI studies were obtained from scans over a one-month period of patients without clinical history or arthroscopic or MRI evidence of ligament injury.

### 2.2. Imaging Methods

**Figure 1:**
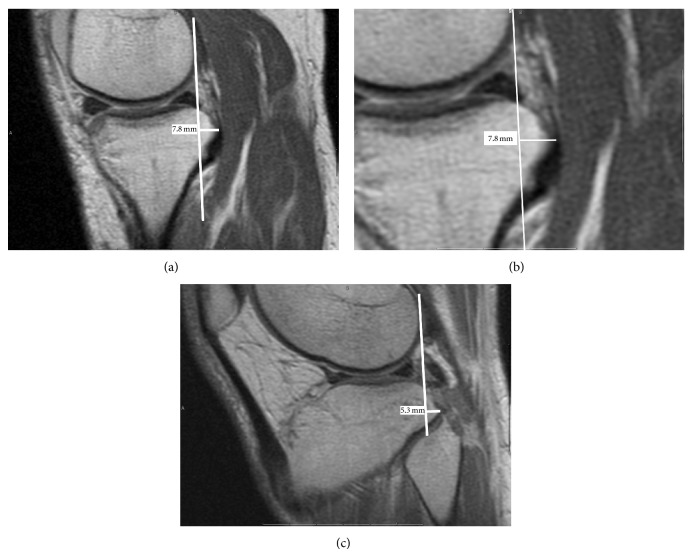
Measurement technique for tibial translation on knee MRI. Magnetic resonance images of the knee in a patient with isolated PCL tear were obtained using sagittal proton density weighted pulse sequence (TR = 3000; TE = 12.168; 1 NEX; 256 × 192 matrix; ETL = 8; 3.00 mm slice thickness). (a) Midmedial compartment measurement of tibial translation demonstrates posterior (+) tibial translation of 7.8 mm in the presence of a PCL tear. (b) Magnified view of measurement of midmedial compartment. (c) Midlateral compartment measurement of tibial translation demonstrates posterior (+) translation of 5.3 mm.

All images were performed on a 1.5 T magnet, using our institutional standard knee MRI protocol. Sagittal PD and fat suppressed T_2_-weighted fast spin-echo imaging, coronal T_1_-weighted and fat suppressed T_2_-weighted fast spin-echo imaging and axial T_2_-weighted fat suppressed FSE imaging sequences were acquired. Images were obtained with the knee in passive extension. Measurements were performed from the sagittal PD images using the method described by Vahey et al. [2, 3]. Measurements were performed in the midmedial and midlateral compartments independently by two musculoskeletal fellowship trained radiologists for all patients and 18 of 22 controls. On the sagittal image, a tangential line was drawn posterior to the femoral condyle, and another similar line was drawn posterior to the tibia plateau. Perpendicular measurements of the relative anterior or posterior translation were made (Figure 1) for each knee.

### 2.3. Statistical Analysis

Statistical analysis using SPSS (Version 20, IBM Corp.) first examined intraclass correlation coefficient of each measurement for assessment of agreement between readers using a two-way random analysis of absolute agreement with a confidence interval of 95%. Group comparison of midmedial and midlateral compartment measurement differences between knees with PCL tear and normal control knees was ascertained by using a nonparametric Mann-Whitney *U* test. For all analyses, differences were considered to be significant when the *P* value was less than 0.05.

## 3. Results

In total, there were 22 individuals (10 male, 12 female) who met inclusion criteria for normal controls without ligamentous injury and 11 individuals (7 male, 4 female) with PCL tear and an intact ACL on arthroscopy. There was no statistically significant difference in age, sex, or laterality between groups as shown in Table 1. Initial MRI evaluation, arthroscopy findings, and average midmedial and midlateral compartment tibial translation for patients with PCL tear are listed in Table 2. At the time of MRI imaging, 3 of the PCL tears were acute (up to 6 weeks after injury), 1 was subacute (6 weeks to 3 months after injury), and 7 were chronic (greater than 3 months after injury). Five of the 11 PCL tears were precipitated by fall or athletic injury and 6 were the result of MVA.

Two observer values of tibial translation in the midmedial and midlateral compartments were averaged for group comparisons and also examined individually to verify differences by single readers as summarized in Table 3. For both individual and averaged measures, there was statistically significant posterior (+) translation of the tibia in the midmedial compartment measuring +2.93 mm in individuals with PCL tear compared to that for knees with normal MRI findings with a mean translation measurement of +0.03 mm, *P* = 0.002. There was excellent reliability between observers for midmedial compartment measurements with an intraclass correlation coefficient of 0.94. No significant difference between groups was demonstrated for midlateral compartment tibial translation, *P* = 0.355. Excellent reliability was seen for lateral compartment measurements with an intraclass correlation coefficient of 0.96.

## 4. Discussion

Diagnosis of posterior cruciate ligament tears is becoming more important as more indications for surgical reconstruction arise [1]. Indications for reconstruction include solitary PCL tears in young active individuals and patients with bony avulsion injuries, high-grade PCL tears, and PCL tears associated with other ligamentous injuries [4]. Patients with chondromalacia of the patella, meniscal derangement, quadriceps atrophy, or degenerative changes may benefit from PCL reconstruction as well [5]. Motor vehicle accidents and sports injuries account for the majority of PCL tears. Sports injuries are more likely to produce isolated PCL tears. Over 50% of tears present more than one year after injury [6]. Isolated PCL tears have a high propensity to result in cartilage damage, which is reported to occur in the medial compartment in 80% of patients and in the patella in 50% of patients by 5 years following the initial injury [7]. The PCL acts as the primary restraint to posterior translation of the tibia [8]. Passive sagittal laxity in the medial compartment resulting from isolated chronic PCL, tear with fixed posterior subluxation of the medial tibial plateau, has been proffered as an explanation for the increased incidence of osteoarthritis in the medial compartment seen in these patients [9]. Functional integrity of the ligament has been clinically determined by posterior tibial translation, graded with the knee flexed at 90 degrees [10]. Swelling, hemarthrosis, and multiligamentous injury may obfuscate assessment on physical exam [11]. Arthroscopically, laxity of the ligament to probing is diagnostic. At examination, posterior translation in the medial compartment ranges from 7.6 to 12.3 mm at 30° and 90° of flexion, respectively [12]. Posterior translation of the tibia is not seen under normal physiologic, resting conditions. MRI studies have also confirmed no significant posterior subluxation of the normal knee in passive extension [3]. In fact, posterior translation of the tibia is occasionally not seen during physical exam in patients with known PCL tears [13]. This has been attributed to significant swelling about the knee joint, hemarthrosis, and an intact arcuate complex. MRI diagnosis can be obfuscated due to the fact that the majority of PCL tears retain continuity on MRI with reports ranging from 62% to 75% [1, 14]. Therefore, indirect signs play a prominent role in the diagnosis of PCL tear. Indirect signs of PCL tears include increased girth of the vertical portion of the PCL, increased intrasubstance signal, and ligamentous laxity [1]. Increased girth of the PCL and increased signal within the ligament are the most reliable of the indirect signs described to date [1]. Only two of the 34 cases in that series were isolated PCL tears [1]. We hypothesize that posterior translation could serve as an additional indirect sign and would be most useful in isolated PCL tear where posterior translation would not be counteracted by anterior translation from concomitant ACL tear and would not be seen in normal knees. In a study of normal knees, mean anterior (−) and posterior (+) translocation of the knee measure 0.3 mm ± 0.5 mm (±2 standard errors) in the midmedial compartment [3]. Our normal reference data was consistent with that of Vahey et al., and, in the cohort with intact ligaments, tibial translation measured 0.09 mm ± 1.53 mm in the midmedial compartment. This method has been applied previously to ACL tear with 58% of patients having torn ACLs reported to show an anterior translation of the tibia relative to the femur of at least 5 mm [3].

Evaluation of anterior-posterior girth of the vertical segment of the PCL, as proposed by Rodriguez Jr. et al., can be problematic in the acute setting, as edema and heterogeneity of signal may make it difficult to discern the anterior and posterior boundaries of the PCL [1]. This may be a significant problem in using this method, since abnormal intrasubstance signal or fluid signal was observed in all 34 cases [1]. Since measurements of ligament girth were performed on the basis of consensus by three musculoskeletal radiologists also not independently, interobserver reliability and reproducibility of their findings have not been established. The difficulty in obtaining measurements in the acute setting with edema may have manifested as poor interobserver reliability if all three radiologists had rendered measurements independently. In normal practice, three radiologists do not read each single MRI and the ability to translate their results into routine practice is unclear and may not be reproducible in a single reader setting. In the acute setting, posterior translation of the tibia may be more easily measured than ligament girth.

One kinematic study investigated posterior tibial translation on MRI [9]. Six patients with chronic, isolated PCL tear were studied. Weight-bearing images were obtained at 0-, 20-, 45-, and 90-degree flexion and non-weight-bearing images were obtained at 90-degree flexion and again at 90-degree flexion with anterior and posterior drawer testing. Those investigators used the Iwaki method for measurements [15]. Their results were similar to ours. They found no statistically significant difference between normal knees and PCL deficient knees in the lateral compartment. In the medial compartment, an average difference of 10.1 mm of posterior tibial subluxation was seen between PCL deficient and normal patients in 90-degree flexion non-weight-bearing and an average of 8.2 mm was seen with drawer testing. Non-weight-bearing MRI exams showed a difference of 5.8 mm on average in posterior tibial subluxation between the PCL deficient patients and normal controls. Several important differences between our study and their study should be noted. We evaluated posterior subluxation on routine knee MRI under routine conditions with the knee extended without weight-bearing. In the study by Logan et al., the weight-bearing exams would require special scanners and non-weight-bearing scans require special maneuvers and manipulations which are not part of a routine scan [9]. In addition, their cases were chronic, where our cases were both chronic and acute. It is unclear if these maneuvers and stresses could be applied to an acute injury and could produce similar results. Also, their study does not have independent readers, and therefore interobserver reliability was not established.

Our study is the first to establish that posterior translation measurements are a valid indicator of acute PCL tear on MRI and a valid indicator of acute or chronic PCL tear on routine MRI with the knee routinely positioned in passive extension. This investigation constitutes the largest study to assess such MRI measurements for isolated PCL tear with arthroscopically intact ACL. While other previous indirect signs of PCL tear have been reported, ours is the first with interobserver reliability data, which demonstrated excellent interobserver reliability between two independent readers for measuring posterior tibial subluxation in the midmedial compartment. Nevertheless, further investigation in a larger population would better define a reference range for future studies and threshold for abnormal values.

We believe that posterior tibial translation may serve as a valuable and reliable indirect sign in particular for solitary cruciate ligament injury consisting of PCL injury in the setting of intact ACL. Combination tears will likely have arthroscopy due to the concomitant ACL injury and therefore will be detected; however, isolated PCL tears are more likely to remain occult. Patients with isolated PCL tears are potentially at risk of degenerative arthritis that could be mitigated by correction of passive sagittal laxity and posterior tibial subluxation in the medial compartment.

This is the first study to apply posterior translation measurements of the tibia to PCL tear with routine MR imaging without maneuvers or weight-bearing. We provide evidence supporting that posterior tibial translation in the midmedial compartment is a promising indirect sign for isolated PCL tear on routine, clinical MRI imaging with excellent interobserver reliability. Such measurements can be obtained clinically and could be utilized to assist in prompt diagnosis and direct appropriate treatment.

## Figures and Tables

**Table 1:** Group demographics summary.

Patient characteristics	No injury	PCL tear	Statistical significance (*P*)
Number of knees	22	11	
Age (yrs.)			
Range	19–58	17–53	0.236
Mean ± SD	37.1 ± 14.5	29.5 ± 12.1	
Sex			
Male	10	7	0.311
Female	12	4	
Laterality			
Right	15	6	0.257
Left	7	5	

**Table 2:** Imaging findings in PCL injury.

Age	Sex	Timing	Etiology	Initial MRI evaluation	Arthroscopic findings	Medial compartment translation	Lateral compartment translation
20	F	Acute	Motor vehicle accident	Partial tear	Complete PCL tear of AL bundle; partial of PM bundle; grade 1 medial femoral cartilage injury	−3.1	−1.6

46	M	Acute	Fall	High-grade partial tear	Intrasubstance tear of AL bundle; intact PM bundle	2.5	0

53	M	Acute	Motor vehicle accident	Complete tear at tibial	Complete tear of the AL bundle	8	6.3

17	F	Chronic	Athletic injury	High-grade partial tear	Complete PCL tear	1	−4.9

21	M	Chronic	Athletic injury	Complete tear	Complete PCL tear with no residual fibers	4.2	2.1

22	M	Chronic	Athletic injury	Complete tear	Grades 2-3 PCL tear; grade 3 posterolateral corner rotatory laxity	3.7	−1.8

25	F	Chronic	Motor vehicle accident	Complete tear	Complete tear of PM bundle, partial tear of AL bundle; grade 2 chondrosis of inferior pole of patella	0	0

28	F	Chronic	Motor vehicle accident	High-grade partial tear	Near complete tear of AL with partial tear of PM bundle; grade 2 trochlear chondral injury	3.3	−5.4

30	M	Chronic	Motor vehicle accident	Partial tear	Intrasubstance PCL stretch injury; medial tibial plateau grade 1 softening; grade 2 medial collateral ligament injury	5.7	1.5

42	M	Chronic	Motor vehicle accident	High-grade partial tear	Grade 3 PCL tear; grade 3 posterolateral corner injury; medial meniscal fraying; grade 2 patellar chondral injury	5.3	−2.5

20	M	Subacute	Motor vehicle accident	Complete tear at tibial	Complete PCL tear of both AL and PM bundles	1.9	0

AL: anterolateral.

PM: posteromedial.

**Table 3:** Comparison of midmedial and midlateral compartment tibial translation measured on MRI.

Measurements	No injury	PCL tear	Difference	Statistical significance (*P*)
Midmedial compartment tibial translation (mm) ± SD	+0.03 ± 1.37	+2.93 ± 3.00	+2.90	0.002^*^
Reader 1	+0.04 ± 1.46	+2.99 ± 2.95	+2.95	0.001^*^
Reader 2	+0.02 ± 1.60	+2.87 ± 3.15	+2.85	0.006^*^
Midlateral compartment tibial translation (mm) ± SD	−0.17 ± 1.71	−0.57 ± 2.05	−0.75	0.355
Reader 1	−0.16 ± 2.18	−0.52 ± 3.71	−0.67	0.396
Reader 2	−0.21 ± 1.77	−0.63 ± 2.93	−0.83	0.375

^*^Statistically significant difference.

Positive (+) values indicate posterior translation of the tibia relative to the femur.

Negative (−) values indicate anterior translation of the tibia relative to the femur.
